# Review of “Adaptive Optics for Biological Imaging” edited by Joel A. Kubby

**DOI:** 10.1186/1475-925X-13-14

**Published:** 2014-02-11

**Authors:** Gaspard Duchêne

**Affiliations:** 1 Department of Astronomy, Hearst Field Annex B-20, University of California Berkeley, Berkeley, CA 94720-3411, USA; 2 Institut de Planétologie et d’Astrophysique (IPAG), UJF-Grenoble 1/CNRS-INSU (UMR 5274), F-38041 Grenoble, France

**Keywords:** Microscopy, High-resolution imaging, Adaptive optics

## Abstract

**Book details:**

*Adaptive Optics for Biological Imaging*. Edited by Joel A. Kubby; CRC Press (Taylor & Francis Group), Boca Raton, Florida, 2013; 359 pages (AOBI, 2013).

ISBN: 978-1-4398-5018-3 (hardback)

## Book review

### Background

Optical imaging systems, such as microscopes or telescopes, aim to provide as crisp and detailed a view as possible. Unfortunately, most devices do not achieve their full optical power due to aberrations that are introduced by the imaging system itself (e.g., the chromatic aberration introduced by a simple lens), in the object to be imaged (e.g., extensive scattering within individual cells), or in the intervening medium (e.g., atmospheric turbulence when imaging astronomical objects). As a result of these aberrations, the incoming wavefronts are distorted, resulting in blurred images. In the case of microscopy, aberrations result in reduced resolution both laterally and axially. In some cases, the aberrations are modest and simple to correct for with static optical elements. However, in most cases, the aberrations are strong, complex in nature, and/or highly dynamical and the loss in image quality incurred can be both dramatic and variable. Aberrations are so common that they have indeed limited the effective resolving power of microscopes and telescopes for centuries.

A variety of remedial methods have been proposed against aberrations but none is as versatile and powerful as adaptive optics (AO) systems. These systems aim at actively controlling and correcting for aberrations, enabling normal imaging systems to reach their nominal power. The key element of an AO system is a “deformable mirror” that can be controlled to compensate the aberrations induced on the incoming wavefronts. The challenges of building an AO system are extremely serious. While the concept was introduced in the astronomical context over 60 years ago, the first on-sky partial experiments awaited the late 1970s and the first full operating system the late 1980s. Nowadays, AO is a mainstream part of modern astronomy: all existing and planned state-of-the-art telescopes have such a device and low-cost versions are being commercialized for small telescopes.

The properties of typical aberrations affecting biological imaging are extremely different from those faced in the astronomical situation. Still, the tremendous potential of AO in this field was rapidly recognized and the first experiments were conducted as early as the late 1980s, almost concurrently with the first astronomical instruments. Since then, the use of AO has increased steadily, first in the field of retinal imaging and later in microscopy, where in-sample scattering and highly complex aberrations combine to present the steepest challenge. Given the improvement in resolution and increased light return that they enable and the many technological and conceptual breakthroughs that have occurred in recent years, it is safe to assume that AO systems will play an increasingly large role in high-resolution biological imaging for the foreseeable future.

Despite its many successes, some of which are illustrated in the book under review, AO remains a “specialist’s tool” within the broad biological imaging community, much as it was among astronomers until the late 1990s. Besides peer-reviewed publications showcasing the power of the technique, dedicated books that provide in-depth discussions of the principles and practical aspects of AO systems are crucially needed. There are several excellent books already available that attempt to fill this gap, but they are primarily focused on the astronomical situation and discuss the microscopy context as an aside at best. The book under review, which focuses exclusively on the biological imaging aspects of AO and aims at introducing this field to non-experts, is a very successful attempt at filling this glaring gap.

### Specific content

The book is constructed in three sections that tackle in turn the principles, methods and applications of AO for biological imaging, offering a complete view of the current state of AO in this field.

The first section of the book covers the optics principles that are commonly used in discussing AO and imaging devices. In keeping with the dual nature of light, this section contains separate chapters covering the principles of wave optics and geometrical optics, as well as a chapter on the theory of image formation. Wave optics, and in particular the diffraction of light, lies at the foundation of AO. However, the discussion of geometric optics in this book is useful as imaging systems are most frequently designed within this framework. While these chapters offer only a brief introduction to rich fields to which entire books are devoted, they set the stage for the rest of the book. In particular, they present key notions related to the effect of aberrations on the formation of images and the point spread function of an imaging device, two key issues that are central to AO. Since these chapters do not discuss AO at all, they provide an easy entry point to steer a non-specialist reader’s attention to the most important concepts that are developed in the rest of the book, as well as the specific vocabulary that permeates the AO literature.

The second section of the book describes several key aspects of an AO system. The first three chapters of this section are devoted to aberrations in the microscopy context, specifically within the sample itself. Written by the same author, these chapters coherently discuss the effects and origin of aberrations, empirical methods to estimate the amplitude of wavefront aberrations, as well as methods to simulate them numerically in the context of idealized objects. Chapter 7 is arguably the most important chapter of the book. It first describes the diversity of techniques available for the two fundamental operations performed by an AO system: wavefront sensing and wavefront correction (chapter 8 provides a much more detailed discussion of wavefront correcting devices that are used in AO systems). The second half of the chapter discusses actual implementations of these devices in the fields of retinal imaging and microscopy, with a special focus on how AO systems help enhance the power of regular imaging devices. The last chapter in this section presents an end-to-end guide to design an AO system, from the conceptual optical design to an alignment procedure and a detailed troubleshooting list. Even though it only addresses a particular type of AO system, this is an immensely valuable part of the book, one that is often overlooked entirely in other books on AO. Yet, as any optics engineer knows, implementing concepts into a physical system is much harder than it seems when reading “theoretical” books/papers and helpful resources are often impossibly hard to find.

The third part of the book is a collection of chapters, some of which are based largely on peer-reviewed publications, that illustrates the diversity of applications of AO in microscopy. Chapters are grouped in two sub-sections, depending on whether wavefront sensing is direct or indirect. In the latter case, no explicit measurement of the wavefront shape is obtained, and it must be reconstructed from the data obtained with the imaging system, as discussed in some detail in Chapter 10. As illustrated in this section, AO has already been applied to multiple forms of microscopy (confocal microscopy, two-photon microscopy, coherence-gated microscopy, wide-field microscopy), and by a number of independent groups. Undoubtedly, the range of applications for microscopy AO systems will continue to expand and the experiments discussed here will eventually become obsolete, much the same way as the early astronomical AO systems pale in comparison to the capabilities of modern systems. Nonetheless, these chapters are extremely valuable in that they present concrete implementations of the notions introduced in the first two sections of the book. Furthermore, most chapters have dedicated sub-sections addressing practical issues encountered in implementing AO systems and critical discussions of the limitation of existing systems. A reader interested in developing a new AO system will find a wealth of useful information in these chapters.

### Suitability for coursework and research

While the book covers many important topics, it is neither a self-standing textbook nor a complete guide to AO for microscopy. Instead, the book is intended as a field-opening introductory book, providing non-experts - and especially potential new users of AO - with the most important concepts and practical aspects. Graduate students and researcher interested in getting their feet wet with AO for microscopy will find it a very useful resource. The book does a very good job of achieving this goal. However, if one intends to develop a new AO system, or to understand the technical subtleties that are usually left out of peer-reviewed publications, this book needs to be complemented by other readings, in particular other AO books that go into much more detail on the underlying theory and principles of AO, albeit not in the specific context of microscopy. To give only one obvious example, the book under review does not discuss in much detail the control schemes that allow the determination of optimal wavefront correction to introduce to achieve optimal AO performance. Yet, this is a critical and non-trivial element of a functioning AO system. The third section of the book, which focuses on setup-specific applications, provides only a glimpse of the diversity and complexity of the problem.

### Overall discussion

The structure of the book is a gradual immersion in the field of AO for microscopy that is, in my opinion, very appropriate to achieve its goals. The team of authors assembled by the editor is broad and extremely knowledgeable. It includes researchers who have worked with AO systems and/or high-resolution microscopy for the best part of two decades and that are recognized leaders in these fields.

Although this is not a fundamental criticism, the book sometimes suffers from the lack of a concerted effort to use uniform notations across chapters, a common issue for books that are collections of chapters written by independent authors. For instance, the indexing of Zernike modes (used as a basis set to decompose complex wavefront aberrations) varies from one chapter to another, which can be very confusing to the reader. More generally, some of the most common concepts are discussed in multiple chapters, with varying degrees of detail and jargon, whereas the book would be easier to read if they were addressed once and for all in a single chapter. Finally, I was a bit surprised by the decision of bypassing entirely the applications of AO to imaging of the eye. A similar book dedicated to vision science was published a few years ago, but a chapter presenting the lessons learned from that field, which is several years ahead of AO for microscopy, would have been valuable.

Overall, this is an excellent book, which achieves its stated primary goal. It fills a gap in the AO literature and, precisely because of its field-specific approach, it will serve as an important tool to democratize the use of AO in the realm of microscopy. Without reservations, this is a book I would recommend to anyone who wishes to start learning about the principles and potential of AO in this field.

## Competing interests

The author declares that he has no competing interests.
